# RNA sequencing reveals widespread transcriptome changes in a renal carcinoma cell line

**DOI:** 10.18632/oncotarget.24269

**Published:** 2018-01-16

**Authors:** Agata Lichawska-Cieslar, Roza Pietrzycka, Janusz Ligeza, Maria Kulecka, Agnieszka Paziewska, Agata Kalita, Dobrochna D. Dolicka, Mateusz Wilamowski, Katarzyna Miekus, Jerzy Ostrowski, Michal Mikula, Jolanta Jura

**Affiliations:** ^1^ Department of General Biochemistry, Faculty of Biochemistry, Biophysics and Biotechnology, Jagiellonian University, Krakow, Poland; ^2^ Departments of Gastroenterology, Hepatology and Clinical Oncology, Centre of Postgraduate Medical Education, Warsaw, Poland; ^3^ Department of Genetics, Maria Sklodowska-Curie Memorial Cancer Centre and Institute of Oncology, Warsaw, Poland

**Keywords:** MCPIP1, clear cell renal cell carcinoma, transcriptome profile, Regnase-1

## Abstract

We used RNA sequencing (RNA-Seq) technology to investigate changes in the transcriptome profile in the Caki-1 clear cell renal cell carcinoma (ccRCC) cells, which overexpress monocyte chemoattractant protein-induced protein 1 (MCPIP1). RNA-Seq data showed changes in 11.6% and 41.8% of the global transcriptome of Caki-1 cells overexpressing wild-type MCPIP1 or its D141N mutant, respectively. Gene ontology and KEGG pathway functional analyses showed that these transcripts encoded proteins involved in cell cycle progression, protein folding in the endoplasmic reticulum, hypoxia response and cell signalling. We identified 219 downregulated transcripts in MCPIP1-expressing cells that were either unchanged or upregulated in D141N-expressing cells. We validated downregulation of 15 transcripts belonging to different functional pathways by qRT-PCR. The growth and viability of MCPIP1-expressing cells was reduced because of elevated p21^Cip1^ levels. MCPIP1-expressing cells also showed reduced levels of DDB1 transcript that encodes component of the E3 ubiquitin ligase that degrades p21^Cip1^. These results demonstrate that MCPIP1 influences the growth and viability of ccRCC cells by increasing or decreasing the transcript levels for proteins involved in cell cycle progression, protein folding, hypoxia response, and cell signaling.

## INTRODUCTION

Clear cell renal cell carcinoma (ccRCC) is the most frequent kidney cancer, which is highly vascularized and characterized by malignant renal epithelial cells with clear cytoplasm. Deletion of the short arm of chromosome 3 that includes the von Hippel Lindau tumor suppressor (VHL) gene correlates with increased expression and activity of HIF-1α and HIF-2α in 90% of the ccRCC patient samples [1]. This is because the VHL protein is part of an active E3 ubiquitin ligase complex that targets HIF-1α for ubiquitin-mediated degradation [2]. The HIF proteins are polyubiquitinated and targeted for degradation during normoxia. However, they accumulate in the nucleus during hypoxia and drive the expression of genes that regulate glycolysis, angiogenesis, and metastasis [3].

HIF-2α is negatively regulated during hypoxia by Monocyte Chemoattractant Protein-induced protein 1 (*MCPIP1*) in the ccRCC cell line, Caki-1 [4]. Moreover, exogenous expression of HIF2-α in Caki-1 cells decreases MCPIP1 protein levels, thereby indicating a negative feedback loop between these two proteins [4]. MCPIP1, also known as Regnase 1, is an RNase that degrades mRNAs and miRNAs [5–7]. The PilT N terminus (PIN) domain is essential for the endonucleolytic activity of MCPIP1 [5, 8]. MCPIP1 negatively regulates expression of various pro-inflammatory cytokines such as IL-6 or IL-1β [5, 8, 9] and also NF-κB or AP-1 transcription factors [10,11]. Furthermore, ectopic overexpression of wild-type MCPIP1 downregulates VEGFA and GLUT1 transcripts that are induced during hypoxia [4]. However, regulation of the whole transcriptome of ccRCC cell line by MCPIP1 has not been studied.

Low MCPIP1 levels have been reported in neuroblastoma [12], breast cancer [13] and ccRCC [4,14]. MCPIP1 inhibits growth of ccRCC and neuroblastoma cell lines [4, 12] by enhancing the decay of anti-apoptotic gene transcripts, including Bcl2L1, Bcl2A1, RelB, Birc3, and Bcl3 [13] and negatively regulating the rate of metabolism and angiogenesis [4]. Furthermore, MCPIP1 regulates the secretion of VEGF, IL-8, and CXCL12, which are factors that promote chemotaxis of microvascular endothelial cells, phosphorylation of VE-cadherin, and increased vascular permeability [14]. *In vitro* and *in vivo* studies show that downregulation of MCPIP1 is associated with epithelial to mesenchymal transition (EMT) and progression of ccRCC [14]. Therefore, in this study, we investigated the role of MCPIP1 in global transcriptional regulation by performing RNA-Seq analysis of Caki-1 cells that overexpress wild type or RNase-deficient MCPIP1 proteins.

## RESULTS AND DISCUSSION

### Global transcriptome changes in Caki-1 cells expressing wild type or mutant MCPIP1

**Figure 1:**
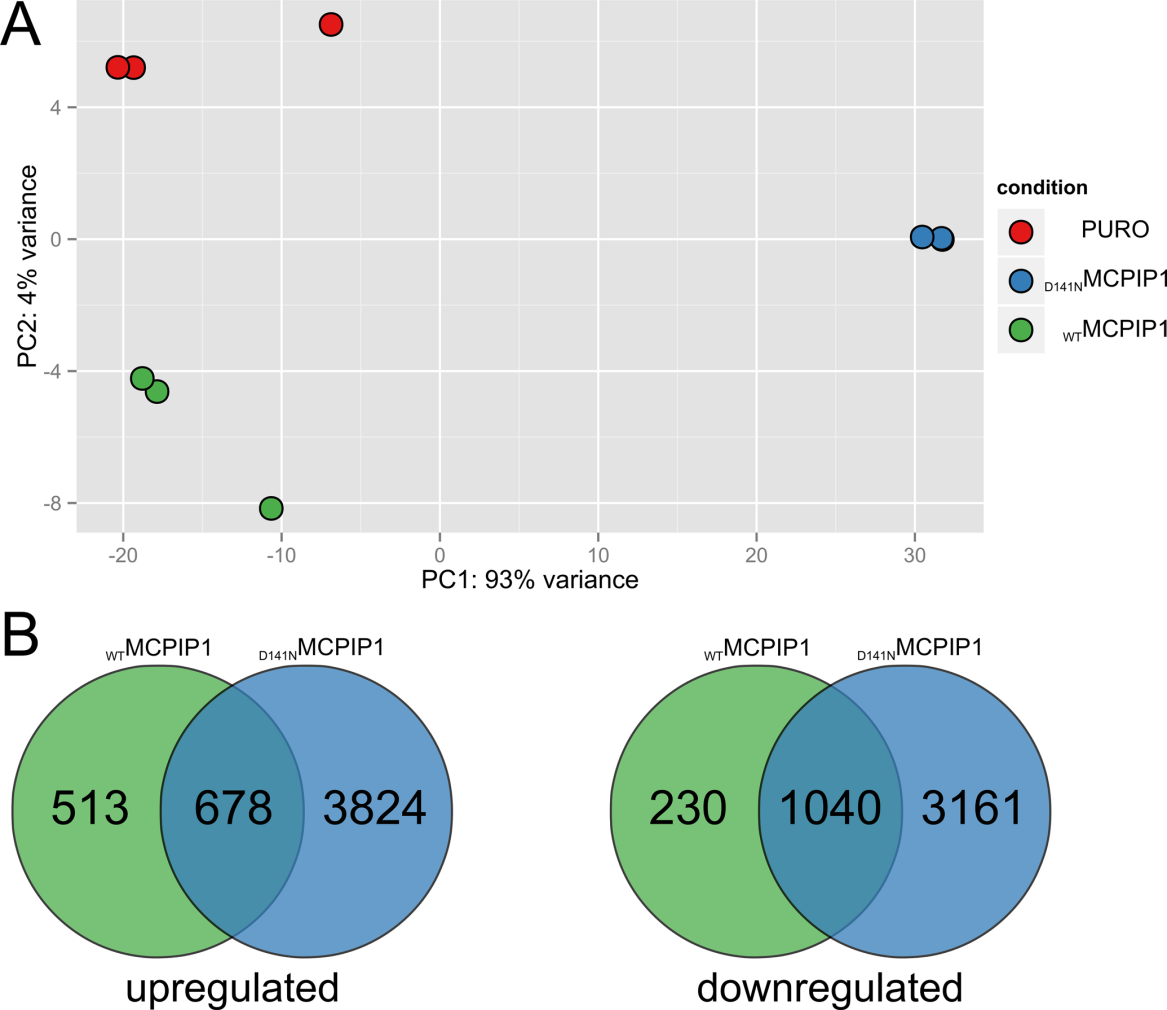
RNA-Seq analysis of global transcriptome changes based on MCPIP1 expression (**A)** Principal component analysis (PCA) of RNA-Seq datasets and (**B**) Venn diagrams show the number of differentially expressed transcripts (adj. *P*-value < 0.05) in the MCPIP1 and D141N cells relative to the PURO cells (MCPIP1 vs. PURO and D141N vs. PURO datasets). PCA is based on the abundances of all transcripts detected in RNA-Seq analysis.

To characterize global transcriptome changes upon MCPIP1 overexpression, we generated Caki-1 cell lines expressing doxycycline-inducible wild-type (MCPIP1) or mutant MCPIP1 (inactivated PIN domain; D141N) using lentiviral vectors. Caki-1 cells transduced with a control lentiviral vector (PURO) were used as control. We performed RNA-Seq analysis of RNA isolated from MCPIP1, D141N and PURO cells, which were grown in media containing puromycin for 10 days. Principal component analysis (PCA) demonstrated differential gene expression in all the 3 cell types (Figure 1A). We performed pairwise comparison of gene expression in MCPIP1 and D141N samples against PURO (adj. p. value < 0.05) and showed that 1189 and 4500 transcripts were upregulated and 1270 and 4201 transcripts were downregulated in MCPIP1 and D141N cells, respectively (Figure 1B, Supplementary Table 1). This accounts for 11.6% and 41.8% of the global transcriptome for MCPIP1 and D141N, respectively, because the AmpliSeq-based RNA-Seq covers 20812 human transcripts.

**Figure 2:**
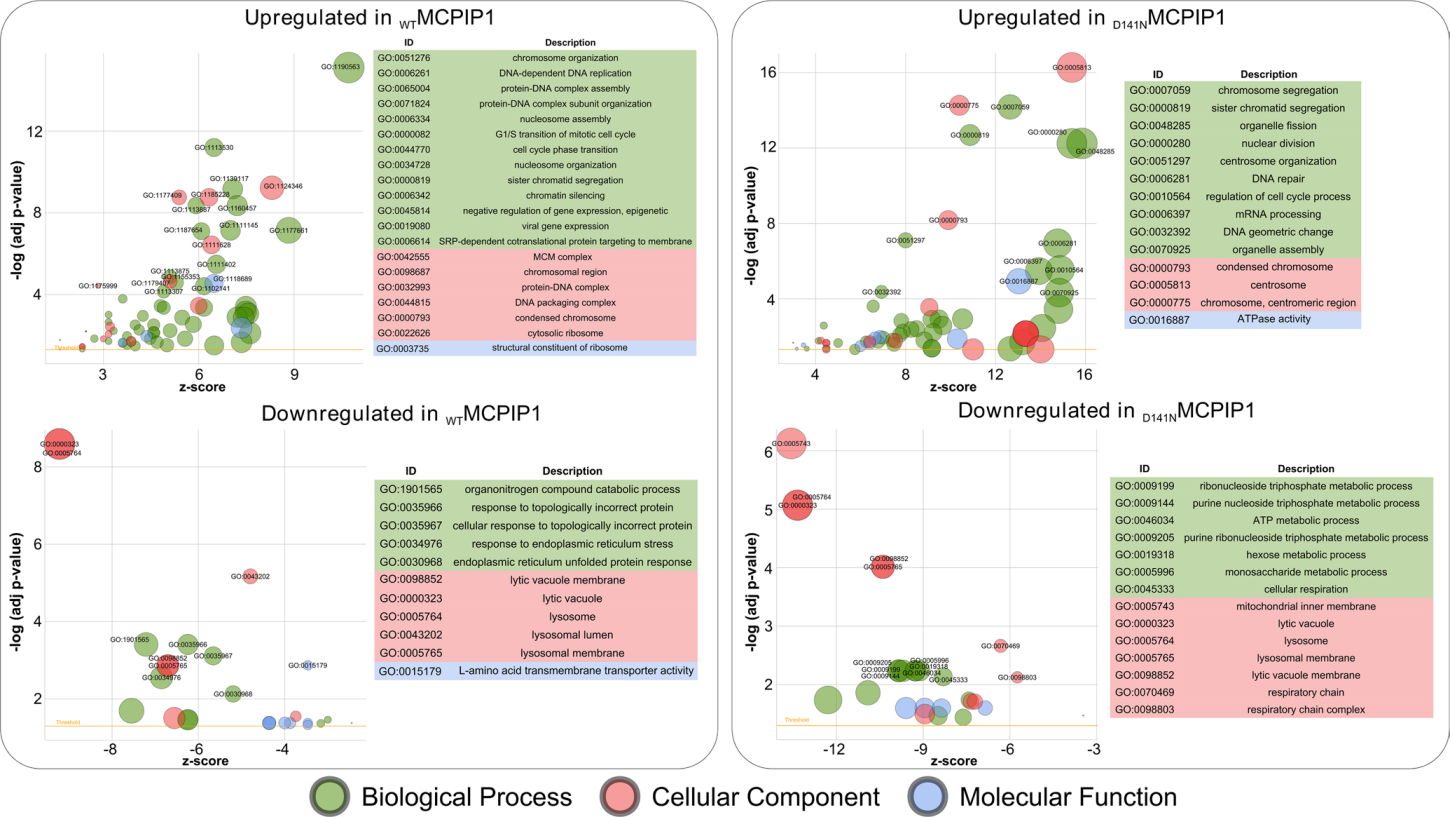
Bubble plot of significantly enriched GO terms in the MCPIP1 vs. PURO and D141N vs. PURO comparative datasets The differentially regulated genes were based on the cut-off set at adj. *p*-value of 10^-4^ and 10^-2^ for upregulated and downregulated genes, respectively. The y-axis represents the -log10 (*p*-value) and the x-axis represents the z-score (computed with GOplot R-package). The area of the displayed circles is proportional to the number of genes assigned to the term in the analysis. The threshold indicates adj. *p* value = 0.05

We performed Gene Ontology (GO) and KEGG enrichment analysis on the differentially expressed genes in the MCPIP1 vs. PURO and D141N vs. PURO groups. The upregulated genes in the MCPIP1 vs. PURO group were enriched in GO terms belonging to 54 biological processes (BP), 4 molecular function (MF), and 13 cellular component (CC) categories (p-adj. < 0.05; Figure 2, Supplementary Table 2). The upregulated genes in the D141N vs. PURO group belonged to 40 BP, 10 MF and 17 CC categories (p-adj. < 0.05; Figure 2 and Supplementary Table 3). The downregulated genes in the MCPIP1 vs. PURO group were enriched in 10 BP, 9 MF, and 7 CC functional categories, whereas the downregulated genes in the D141N vs. PURO group were enriched in 13 BP, 4 MF and 10 CC categories (p-adj. < 0.05; Figure 2, Supplementary Tables 2 and 3). The biological processes upregulated in MCPIP1 and D141N cells (p-adj. < 0.0001) were common in both cell lines and involved in cell cycle, cell division, DNA replication, and DNA repair, whereas those downregulated (p-adj. < 0.01) were distinct in MCPIP1 and D141N cells, and were involved in endoplasmic reticulum stress and nucleotide metabolism, respectively. Moreover, transcripts associated with lysosomes were downregulated in both MCPIP1 and D141N cells.

KEGG enrichment analysis shows 8 upregulated and 2 downregulated pathways in MCPIP1 cells, whereas, 12 upregulated and 7 downregulated pathways were observed in D141N cells (p-adj. < 0.05; Table 1, Supplementary Figure 1, Supplementary Tables 2 and 3). Cell cycle was the top upregulated KEGG pathway, whereas lysosomal regulation was the most downregulated KEGG pathway in MCPIP1 and D141N cells. We additionally analyzed the differentially expressed genes between MCPIP1 and D141N overexpressing cells. GO analysis showed that upregulated genes belonged to 37 BP, 11 MF and 11 CC GO terms, whereas the downregulated genes belonged to 50 BP, 12MF and 20 CC GO terms. KEGG enrichment analysis showed 8 upregulated and 16 downregulated pathways in MCPIP1 cells than in D141N cells. Both GO and KEGG analyses showed that wild-type MCPIP1 downregulated cell cycle, DNA repair and endoplasmic reticulum protein processing (Supplementary Table 4).

**Table 1:** KEGG pathways overrepresented among genes differentiating control and MCPIP1 and D141N cells

	ID	Description	GeneRatio	BgRatio	*p* value	*q* value	Count
**MCPIP1 vs Control**	Upregulated						
	**hsa03030**	**DNA replication**	**18/481**	**32/4312**	**5.61E-10**	**1.45E-07**	**18**
	hsa05322	Systemic lupus erythematosus	28/481	74/4312	1.44E-09	1.86E-07	28
	**hsa04110**	**Cell cycle**	**34/481**	**113/4312**	**2.45E-08**	**2.10E-06**	**34**
	hsa05034	Alcoholism	34/481	125/4312	3.74E-07	2.41E-05	34
	hsa03010	Ribosome	32/481	118/4312	8.97E-07	4.63E-05	32
	hsa04115	p53 signaling pathway	16/481	55/4312	2.11E-04	9.08E-03	16
	**hsa03430**	**Mismatch repair**	**8/481**	**20/4312**	**8.42E-04**	**3.10E-02**	**8**
	**hsa03420**	**Nucleotide excision repair**	**12/481**	**40/4312**	**9.62E-04**	**3.10E-02**	**12**
	Downregulated						
	**hsa04142**	**Lysosome**	**29/517**	**103/4312**	**5.63E-06**	**1.52E-03**	**29**
	hsa04610	Complement and coagulation cascades	13/517	32/4312	3.63E-05	4.90E-03	13
**D14N vs Control**	Upregulated						
	**hsa04110**	**Cell cycle**	**76/1574**	**116/4752**	**5.18E-13**	**1.45E-10**	**76**
	hsa04114	Oocyte meiosis	58/1574	95/4752	1.65E-08	2.30E-06	58
	hsa04120	Ubiquitin mediated proteolysis	63/1574	119/4752	5.03E-06	4.68E-04	63
	hsa04914	Progesterone-mediated oocyte maturation	41/1574	78/4752	2.76E-04	1.93E-02	41
	hsa04141	Protein processing in endoplasmic reticulum	70/1574	151/4752	4.07E-04	2.27E-02	70
	**hsa03430**	**Mismatch repair**	**15/1574**	**22/4752**	**7.94E-04**	**3.69E-02**	**15**
	**hsa03420**	**Nucleotide excision repair**	**24/1574**	**42/4752**	**1.11E-03**	**3.87E-02**	**24**
	hsa03013	RNA transport	62/1574	135/4752	1.15E-03	3.87E-02	62
	hsa03018	RNA degradation	35/1574	68/4752	1.25E-03	3.87E-02	35
	hsa03460	Fanconi anemia pathway	25/1574	45/4752	1.53E-03	4.01E-02	25
	hsa03015	mRNA surveillance pathway	38/1574	76/4752	1.58E-03	4.01E-02	38
	hsa03040	Spliceosome	57/1574	124/4752	1.75E-03	4.06E-02	57
	Downregulated						
	**hsa04142**	**Lysosome**	**63/1564**	**113/4752**	**3.61E-07**	**1.03E-04**	**63**
	hsa01230	Biosynthesis of amino acids	33/1564	54/4752	1.79E-05	2.55E-03	33
	hsa04932	Non-alcoholic fatty liver disease	64/1564	127/4752	2.71E-05	2.58E-03	64
	hsa01200	Carbon metabolism	47/1564	91/4752	1.48E-04	1.05E-02	47
	hsa00190	Oxidative phosphorylation	52/1564	104/4752	1.96E-04	1.12E-02	52
	hsa05012	Parkinson’s disease	53/1564	108/4752	3.13E-04	1.49E-02	53

Terms common for both comparisons are bolded. ID – KEGG identifier, GeneRatio – the ratio of number of differentiating genes in a given pathway to the number of differentiating genes with KEGG identifier ; BgRatio – the ratio of number of not differentiating genes in a given pathway to the number of expressed genes with KEGG identifier; *p*-value - *p*-value in hypergeometric test; *q*-value - *p*-value after FDR correction; Count – number of differentiating genes contributing to a given pathway.

Thus, the RNA-Seq analysis revealed widespread changes in transcript levels in both MCPIP1 and D141N cells. These changes were more pronounced in D141N cells. The functional analysis with GO and KEGG databases demonstrated overlapping and distinct cellular processes for cell lines overexpressing wild type and mutated MCPIP1 protein.

### Putative MCPIP1 targets

Next, we analyzed 219 genes that were were downregulated in MCPIP1 cells based on RNA-Seq analysis as possible targets of MCPIP1 RNase. Among these, 183 genes were unchanged in D141N cells (p-adj. < 0.05), whereas, the remaining 36 were upregulated in D141N cells (fold change > 1.5 and p-adj. < 0.05; Supplementary Table 1). On the basis of our functional analysis and on the literature data we selected 15 out of these 219 genes for further validation. These were involved in protein folding, cell cycle regulation, hypoxia response and cell signaling (Table 2). We verified the expression of these 15 selected genes by quantitative real time PCR (qRT-PCR) using the primer sequences listed in Supplementary Table 5.

**Table 2:** List of 15 transcripts selected for validation based on the RNA-Seq analysis

Gene Symbol	Description	Function/Characteristics	MCPIP1 vs PURO		D141N vs PURO	
			adj.*p*val	FC	adj.*p*val	FC
**AGR2**	Anterior Gradient 2	• **Protein folding** and maturation in the endoplasmic reticulum • **Hypoxia**-induced expression	**0.00733**	**0.47**	**0.01477**	**1.59**
**DDB1**	Damage Specific DNA Binding Protein 1	• Involved in degradation of **cell cycle** regulators • DNA Repair	**0.01228**	**0.69**	0.52111	1.09
**ENPP2**	Ectonucleotide Pyrophosphatase/ Phosphodiesterase 2	• **Hypoxia**-induced expression • Promoting cancer cell metastasis and angiogenesis	**0.03466**	**0.46**	**0.00479**	**2.29**
**FRAT1**	Frequently Rearranged In Advanced T-Cell Lymphomas 1	• **Signal Transduction** • Regulating Wnt signaling	**0.00631**	**0.42**	0.70815	0.90
**GPRC5B**	G Protein-Coupled Receptor Class C Group 5 Member B	• **Signal Transduction** • **Hypoxia**-induced expression • Associated with lipid metabolism	**0.01287**	**0.58**	0.95273	1.01
**HSPA5**	Heat shock 70kDa protein 5	• **Protein folding** and maturation in the endoplasmic reticulum • **Hypoxia**-induced expression	**0.00042**	**0.59**	0.82536	0.96
**MMP2**	Matrix Metallopeptidase 2	• **Hypoxia**-induced expression • Promoting cancer cell metastasis and angiogenesis	**0.01807**	**0.42**	**0.00003**	**3.31**
**NDRG1**	N-Myc Downstream Regulated 1	• **Hypoxia**-induced expression	**0.00000**	**0.36**	0.05544	0.76
**NDRG2**	NDRG Family Member 2	• **Hypoxia**-induced expression	**0.00924**	**0.54**	0.28197	0.80
**NGEF**	Neuronal Guanine Nucleotide Exchange Factor	• **Signal Transduction** • Associated with lipid metabolism	**0.00030**	**0.41**	0.90357	0.98
**PLOD2**	Procollagen-Lysine,2-Oxoglutarate 5-Dioxygenase 2	• **Hypoxia**-induced expression • Promoting cancer cell metastasis	**0.00244**	**0.48**	**0.00000**	**5.00**
**RIPK4**	Receptor Interacting Serine/Threonine Kinase 4	• **Signal Transduction** • Regulating Wnt signaling	**0.00733**	**0.45**	0.86098	0.94
**SGK2**	Serine/Threonine Kinase 2	• **Signal Transduction** • Regulating sodium transporters	**0.00200**	**0.49**	0.09619	1.26
**SPHK1**	Sphingosine Kinase 1	• **Hypoxia**-induced expression • **Signal Transduction** • Promoting cancer cell metastasis and angiogenesis	**0.00000**	**0.32**	0.23539	0.83
**TSC22D3**	TSC22 Domain Family Member 3	• **Signal Transduction** • Interfering with NF-κB signaling • Regulating sodium transporters	**0.00430**	**0.52**	0.77541	0.95

The fold change (FC) and adjusted *P*-values for the MCPIP1 vs. PURO and D141N vs. PURO comparisons are provided. The full list of transcripts is shown in Supplementary Table 1.

### PIN domain is important for viability and proliferation of Caki-1 cells

**Figure 3:**
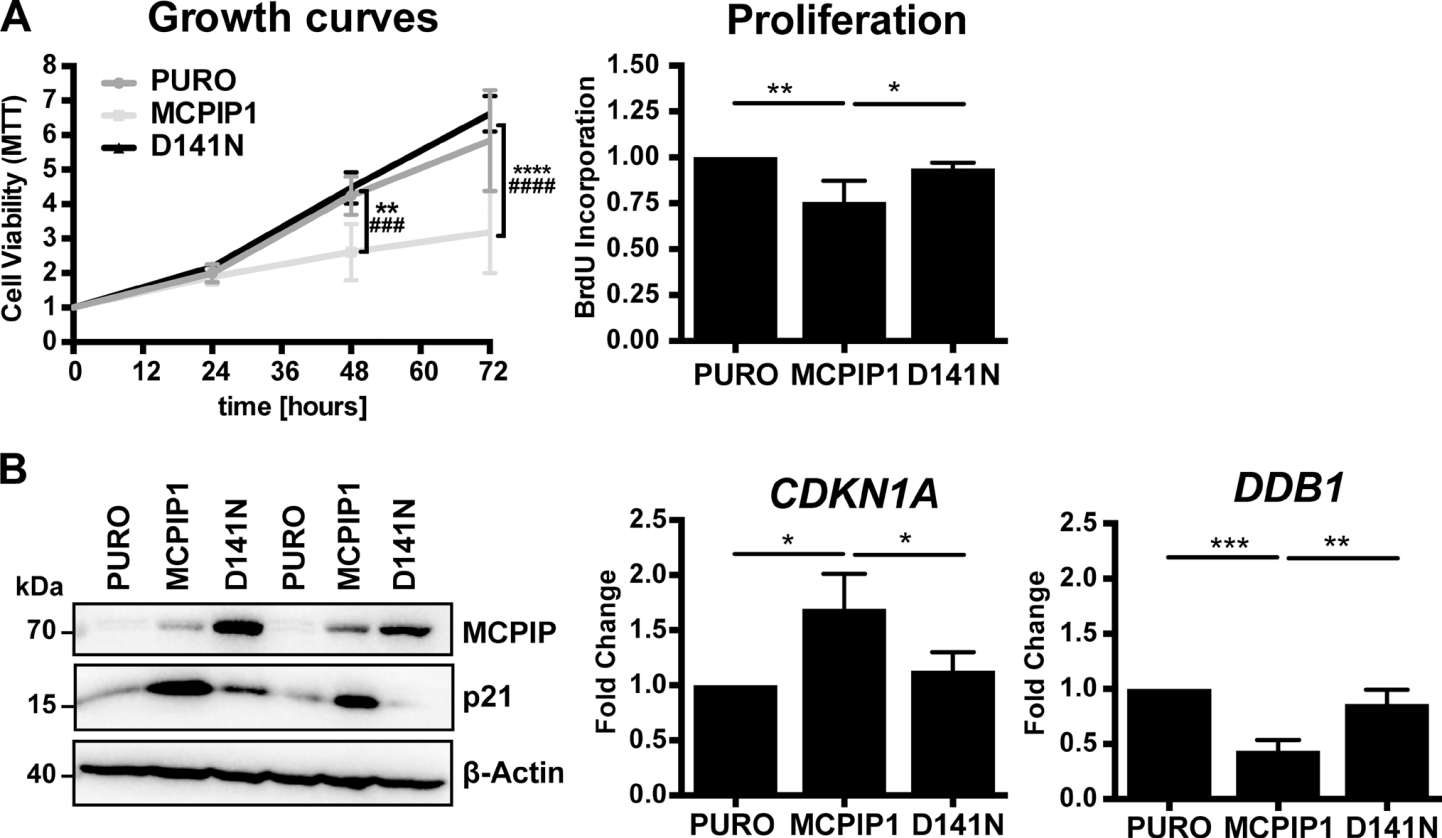
MCPIP1 overexpression decreases growth of Caki-1 cells (**A**) Graphical representation of cell viability, proliferation and growth of PURO, MCPIP1 and D141N cells. Cell growth and viability was measured by the MTT assay at 24, 48 and 72 h in the 3 cell lines after induction with doxycycline for 24 h. Cell proliferation was measured by BrdU incorporation for 8 h in the three cell lines after induction with doxycycline for 24 h. (**B**) Representative western blots show MCPIP1 and p21 expression in PURO, MCPIP1 and D141N cells induced with doxycycline for 24 h. β-Actin is used as a loading control. Histograms show CDKN1A and DDB1 transcript levels relative to RPS13 transcript levels in PURO, MCPIP1 and D141N cells induced with doxycycline for 24 h. Blots represent two out of three identical experiments. Graphs represent mean ± SD from three (B) or four (A) independent experiments. The *p*-values were estimated by one-way ANOVA followed by Tukey’s HSD tests (^*^p < 0.05; ^**^p < 0.01; ^***^p < 0.001; ^****^p < 0.0001). Note: For the cell growth analysis the two-way ANOVA followed by Tukeys’s independent test was used (^*^ relates to PURO vs. MCPIP1 comparison and # relates to MCPIP1 vs. D141N comparison).

Previous reports show that overexpression of wild-type MCPIP1 in Caki-1 cells decreases cell survival and proliferation [4]. We confirmed that the MCPIP1 cells showed decreased confluence than in PURO and also in D141N cells. MTT assay also showed that MCPIP1 cells were less viable than PURO and D141N cells (Figure 3A). BrdU incorporation assay showed decreased proliferation of MCPIP1 cells than in PURO and D141N cells (Figure 3A). These results suggest that MCPIP1 regulates proliferation and viability of Caki-1 cells.

### MCPIP1 regulates transcripts of cell cycle regulatory genes

We investigated growth inhibition in MCPIP1 cells by analyzing expression of some cell cycle genes. MCPIP1 cells showed increased expression of the p21^Cip1^ (CDKN1A) protein and mRNA than in PURO and D141N cells (Figure 3B). The p21^Cip1^ protein belongs to the Cip/Kip family of inhibitors and blocks cell cycle by inhibiting G1/S and S-phase Cyclin-Cdks (Cyclin D, E and A) [15]. During S phase, p21^Cip1^ degradation is regulated by the activity of Cul4-DDB1-Cdt2 E3 ligase [16]. In our RNA-Seq analysis, DDB1 (Damage Specific DNA Binding Protein 1) transcript levels were reduced in MCPIP1 cells than in PURO and D141N controls (Table 2 and Supplementary Table 1). QRT-PCR analysis showed that DDB1 mRNA levels were reduced by 2.3-fold and 2-fold in MCPIP1 cells than in PURO and D141N cells (Figure 3B). Therefore, we postulate that lower levels of DDB1 will decrease the growth of MCPIP1 cells by reducing p21^Cip1^ degradation. Cang *et al.* showed that conditional knockout of DDB1 in mouse brain blocks the cell cycle and promotes apoptosis [17]. DDB1 knockdown upregulates both p21 protein and mRNA levels, thereby suggesting that regulation of p21 is complex [18].

We also observed that CDT1 (Chromatin Licensing and DNA Replication Factor 1) mRNA levels are upregulated in MCP1P cells (adj. *p*-value = 3.076E-08 as compared to PURO) and unchanged in D141N cells (Supplementary Table 1). CDT1 is negatively regulated by Cul4-DDB1-Cdt2 complex [17, 19, 20]. It is required for the assembly of pre-replicative complexes (pre-RC) at origins of replication in G1 phase [21]. In human cells, elevated Cdt1 levels promotes re-replication, prevents entry into mitosis and inhibits cell growth by activating Chk1/Chk2 kinases and p21^Cip1^ [22, 23]. This suggests that MCPIP1 overexpression inhibits cell cycling and growth by upregulating the p21^Cip1^ cell cycle inhibitor in cells.

### MCPIP1 regulates transcripts of factors involved in ER protein misfolding response

MCP1P1 overexpression downregulates transcripts that encode proteins involved in protein folding in the ER (Figure 2, Table 2, Supplementary Table 1). Chaperones facilitate protein re-folding and target misfolded proteins for degradation [24]. The protein folding machinery is dysregulated in MCPIP1 cells, which results in the accumulation of misfolded proteins. This may inhibit their proliferation [25]. We validated expression of HSPA5 and AGR2 transcripts, which are involved in protein folding and are downregulated in MCPIP1 cells (Table 2 and Supplementary Table 1).

**Figure 4:**
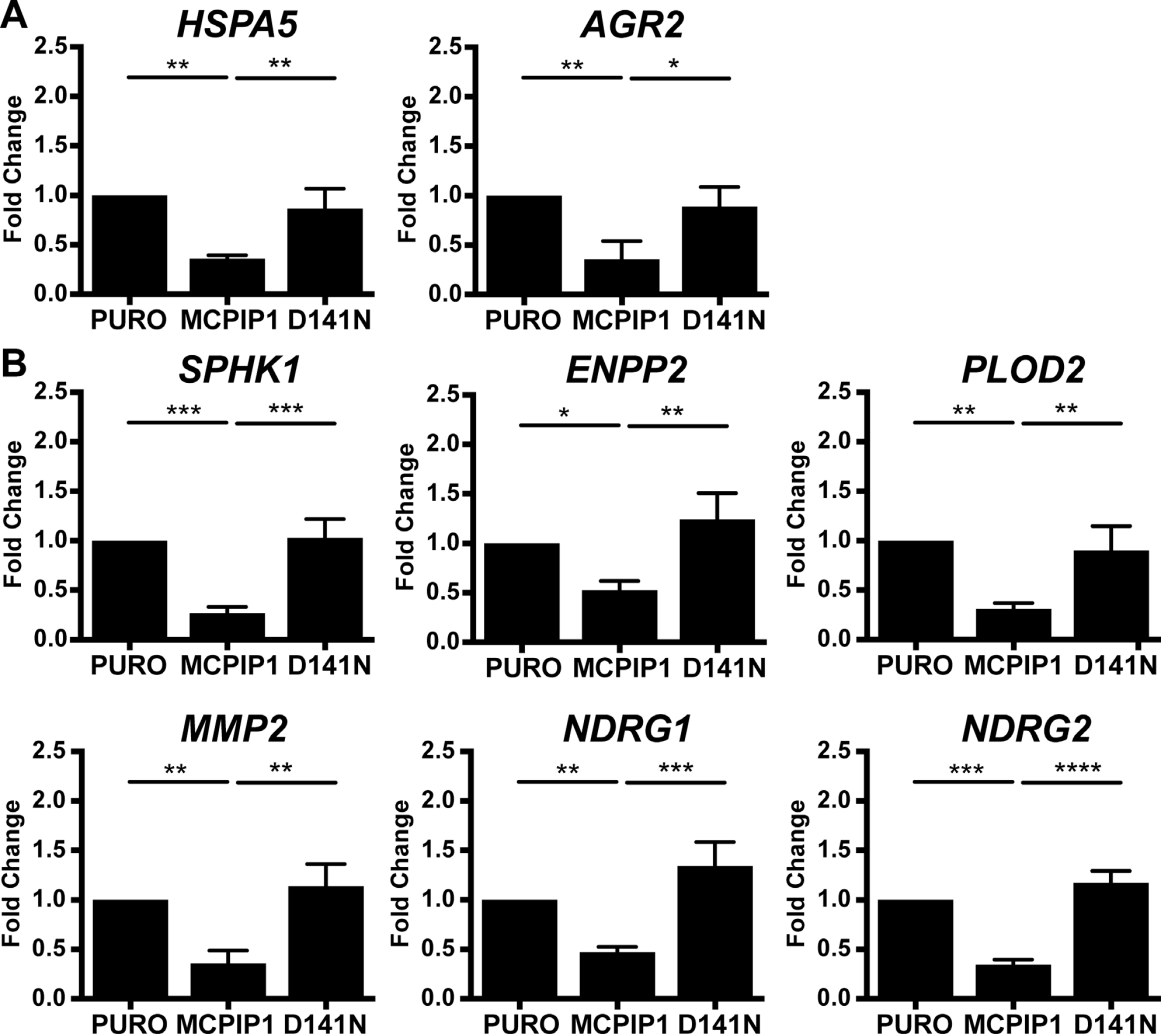
MCPIP1 decreases transcripts of protein folding and hypoxia response genes (**A**) QRT-PCR analysis of HSPA5 and AGR2 mRNA levels in PURO, MCPIP1 and D141N cells induced with doxycycline for 24 h. (**B**) QRT-PCR analysis of SPHK1, ENPP2, PLOD2, MMP2, NDRG1 and NDRG2 mRNA levels in PURO, MCPIP1 and D141N cells induced with doxycycline for 24 h. Transcript levels are normalized to RPS13 mRNA levels as control. Graphs represent mean ± SD of three independent experiments. The *p*-values were estimated by one-way ANOVA followed by Tukey’s HSD test (^*^ *p* < 0.05; ^**^p < 0.01; ^***^p < 0.001; ^****^p < 0.0001).

HSPA5 is a member of the heat shock protein 70 (HSP70) family, which is induced during various stress conditions such as hypoxia to regulate protein folding and assembly in the ER [26]. QRT-PCR analysis showed that HSPA5 mRNA levels in MCPIP1 cells were reduced by 2.8-fold and 2.4-fold than in PURO and D141N cells, respectively (Figure 4A).

Anterior gradient 2 (AGR2) is a member of the family of disulphide isomerases that regulate protein folding, maturation and secretion of proteins in the ER and is regulated by HIF-1α [27]. AGR2 mRNA levels in MCPIP1 cells were 2.8-fold and 2.5-fold lower than in PURO and D141N cells, respectively (Figure 4A). AGR2 specifically binds and stabilizes HIF-1α decreasing its proteasomal degradation [28]. Moreover, AGR2 overexpression is associated with survival, invasion and epithelial-mesenchymal transition of many cancers; for instance head and neck squamous cell carcinoma, pancreatic and breast cancers [29–31]. These data suggest that factors such as HSPA5 and AGR2 protect the cancer cells that reside in a hypoxic environment from ER-stress induced apoptosis.

It was shown that in hypoxic conditions in mouse beta cells downregulation of the protein folding machinery in the ER promotes cell death [32]. Here, we postulate that low MCPIP1 levels promotes growth and progression of ccRCC cells by upregulating factors such as HSPA5 and AGR2, which are involved in protein folding and secretion in the ER.

### MCPIP1 downregulates hypoxia-response transcripts

We have already showed that MCPIP1 overexpression negatively regulates vascular endothelial growth factor (VEGF) [4], a well-known pro-angiogenic factor that is upregulated in many tumors [33–35]. In RNA-Seq we consistently found VEGF to be significantly downregulated (adj. *p*-value = 4.69E-04 as compared to PURO) in MCPIP1 overexpressing cells (Supplementary Table 1). Although the role of MCPIP1 in hypoxia and angiogenesis has been previously reported, the molecular mechanism is not fully elucidated.

As reported above, HSPA5 and AGR2 are two MCPIP1-regulated ER proteins that play an important role in hypoxia. Therefore, we validated a subset of hypoxia-related genes that are downregulated in MCPIP1 cells, but remained unchanged or upregulated in D141N cells (Table 2 and Supplementary Table 1).

Sphingosine Kinase 1 (SPHK1) is classified under the *organonitrogen compound catabolic process*, one of the top BP term for transcripts that are downregulated in MCPIP1 vs. PURO group (Figure 2 and Supplementary Table 2). SPHK1 catalyzes phosphorylation of sphingosine to sphingosine-1-phosphate (S1P), which promotes proliferation; meanwhile, sphingosine induces cell growth arrest and apoptosis (reviewed in [36, 37]). During hypoxia, SPHK1 regulates expression of HIF1α and HIF2α [38, 39]. SPHK1 overexpression promotes angiogenesis by inducing VEGFA and MMP2 expression in many tumors [40, 41]. SPHK1 transcript levels were reduced by 3.7-fold and 3.8-fold in MCPIP1 cells than in PURO and D141N cells, respectively (Figure 4B). Therefore, we postulate that low MCPIP1 expression in ccRCC cells increase S1P levels by enhancing SPHK1, which results in higher proliferation rates.

RNA-Seq data also showed that ENPP2 (Ectonucleotide Pyrophosphatase/ Phosphodiesterase family member 2) transcript levels were significantly downregulated in MCPIP1 cells (Supplementary Table 1 and Table 2). ENPP2 or autotaxin functions as a phosphodiesterase and phospholipase that generates lysophosphatidic acid (LPA), which stimulates cell proliferation and chemotaxis [42]. ENPP2 is upregulated in several carcinomas and promotes angiogenesis [42, 43]. Hypoxia stimulates expression of ENPP2 as well as production of LPA [44]. Moreover, LPA induces HIF1α expression in colon cancer cells at the transcriptional level [45]. ENPP2 transcript levels were reduced in MCPIP1 cells by 1.9-fold and 2.4-fold than in PURO and D141N cells, respectively (Figure 4B). Since VEGF-A and LPA is a are both proangiogenic factors, our findings suggest that MCPIP1 overexpression inhibits angiogenesis in Caki-1 cells by downregulating VEGFA and ENPP2.

We further showed that the transcript encoding PLOD2 (Procollagen-Lysine 2-Oxoglutarate 5-Dioxygenase 2) was downregulated in MCPIP1 cells by 3.2-fold and 2.9 than in PURO and D141N cells, respectively (Figure 4B). Similarly, transcript encoding MMP2 (matrix metalloproteinase) was also decreased by 2.8-fold and 3.2-fold in MCPIP1 cells than in PURO and D141N cells, respectively (Figure 4B). PLOD2 is an enzyme that catalyzes the hydroxylation of lysyl residues in collagen-like peptides and is involved in extracellular matrix remodeling and hypoxia-induced breast cancer metastasis [46]. PLOD2 expression is induced in hypoxia by HIF1α [46–48]. MMP2 is also activated by hypoxia and facilitates vascular invasion during angiogenesis [49–51]. MMP2 cleaves extracellular matrix proteins and is involved in signal transduction. Hypoxia-driven MMP2 activation promotes cancer progression and metastasis [52, 53]. Zhu *et al.* showed that high MMP-2 expression was associated with poor prognosis and rapid progression of RCC [54]. These results suggest that MCPIP1 modulates extracellular matrix remodeling in angiogenesis and various cancers by regulating expression of factors such as MMP2 and PLOD2.

N-Myc downstream-regulated gene 1 (NDRG1) and NDRG Family Member 2 (NDRG2) are two other hypoxia-related transcripts that are downregulated in MCPIP1 cells. NDRG1 transcript levels were decreased by 2.1-fold and 2.8-fold in MCPIP1 cells than in PURO and D141N cells, respectively (Figure 4B). NDRG1 is involved in stress and hormone responses as well as cell growth and differentiation. NDRG1 is necessary for p53-mediated caspase activation and apoptosis [55]. High NDRG1 expression has shown potential as a prognostic biomarker in many types of cancers [56, 57], including ccRCC [58]. Moreover, NDRG1 expression is differentially regulated during diverse physiological and pathological conditions such as hypoxia, cellular differentiation, heavy metal response and neoplasia [56, 59–61]. NDRG2 mRNA levels were reduced by 2.9-fold and 3.4-fold in MCPIP1 cells than in PURO and D141N, respectively (Figure 4B). NDRG2 suppresses renal cell carcinoma proliferation and invasion [62, 63]. NDRG2 regulation is complex and involves both N-Myc and MCPIP1 [64].

### MCPIP1 negatively regulates transcripts encoding signaling mediators

**Figure 5:**
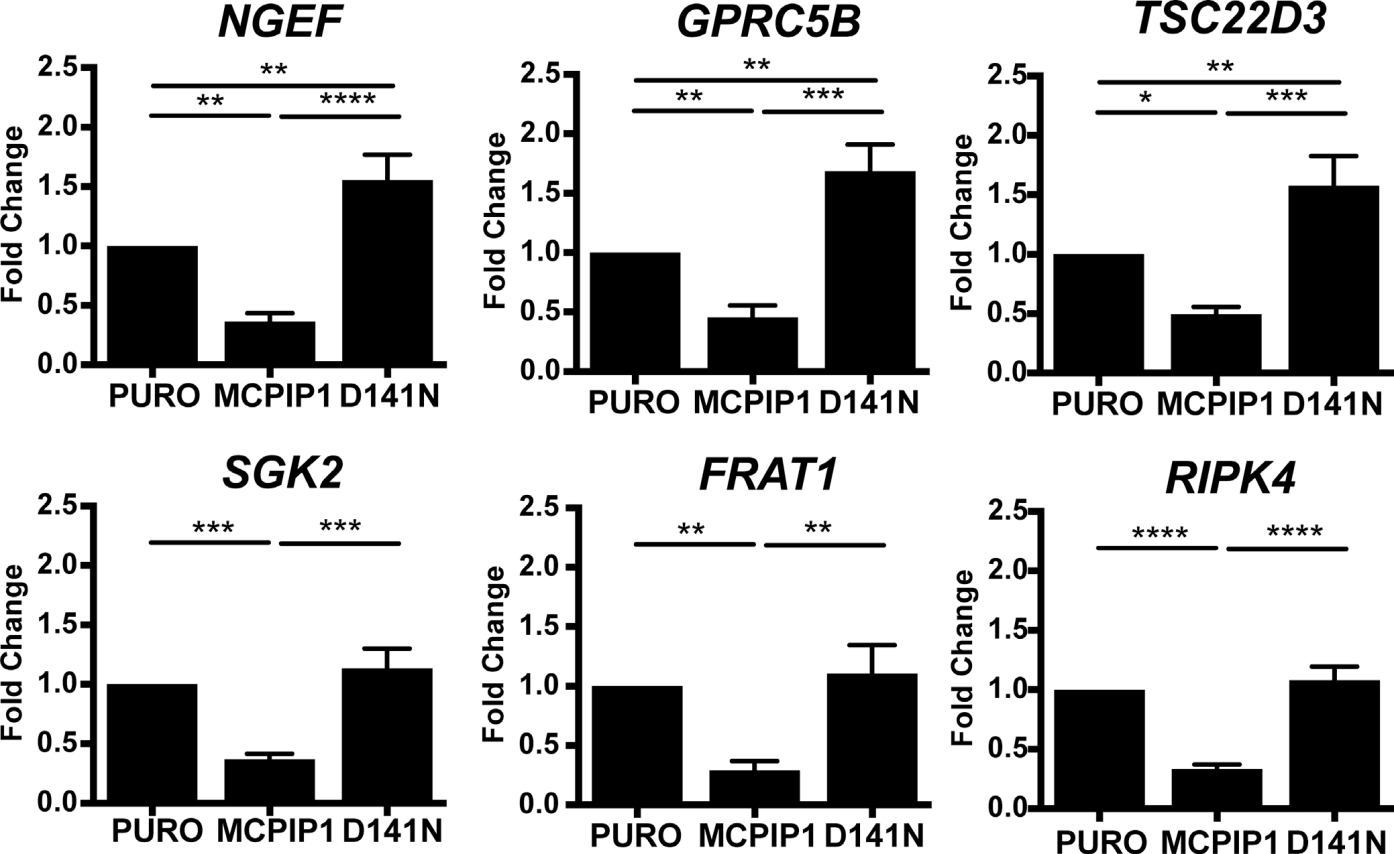
MCPIP1 decreases transcripts of signaling mediators QRT-PCR analysis of NGEF, GPRC5B, TSC22D3, SGK2, FRAT1 and RIPK4 mRNA levels relative to RPS13 in PURO, MCPIP1 and D141N cells induced with doxycycline for 24 h Graphs represent mean ± SD of three independent experiments. The *p*-values were estimated by one-way ANOVA followed by Tukey’s HSD test ((^*^ *p* <0.05; ^**^p <0.01; ^***^p <0.001; ^****^p <0.0001).

Finally, we validated six transcripts that encode signaling pathway mediators, which were downregulated in MCPIP1 cells (Supplementary Table 1; Table 2 and Figure 5). These included the transcript encoding for neuronal guanine nucleotide exchange factor (NGEF) or Ephexin1. *NGEF* gene polymorphism is associated with obesity and adipose tissue content [65, 66]. Moreover, ccRCC is composed of cells whose cytoplasm is filled with lipid droplets [67, 68]. We previously reported the role of MCPIP1 in adipocyte differentiation [69] and that forced expression of MCPIP1 reduces differentiation potential of pre-adipocytes [70]. NGEF mRNA levels were reduced by 2.7-fold and 4.3-fold in MCPIP1 cells than in PURO and D141N cells, respectively (Figure 5). We postulate that changes in NGEF mRNA levels may impact lipid storage in Caki-1 cells.

G-protein coupled receptor family C group 5 member B (GPRC5B) is a putative glutamate G-protein coupled receptor [71–75], which is associated with obesity by promoting adipose inflammation [76–78]. We observed that the GPRC5B mRNA levels in MCPIP1 were reduced by 2.2-fold and 3.7-fold than in PURO and D141N cells, respectively (Figure 5).

TSC22D3 (TSC22 Domain Family Member 3) transcript encodes the glucocorticoid-induced leucine zipper (GILZ) protein, which inhibits adipogenesis by downregulating PPARγ and CEBPα [79]. Moreover, GILZ protein is an important mediator of anti-inflammatory effects in gastric cancer [80–85]. GILZ binds to NF-κB and inhibits its nuclear translocation [86–88]. It mediates anti-inflammatory effects by directly interacting with AP-1 [89] and/or inhibiting upstream signaling pathways by binding to Ras and Raf-1 kinase [90, 91]. Similar to the other hypoxia-related transcripts that are downregulated in MCPIP1 cells, GILZ is induced by hypoxia [92]. Moreover, GILZ is involved in the epithelial Na+ transport by cooperating with SGK2 (Serine/Threonine Kinase 2), which regulates the expression of sodium-coupled transporters that mediate transmembrane transport of important metabolites [93–100]. Figure 5 shows that TSC2DD3 transcript levels were reduced by 2-fold and 3.2-fold in the MCPIP1 cells relative to PURO and D141N cells, respectively (Figure 5). The levels of SGK2 mRNA were 2.7-fold and 3.1-fold lower in MCPIP1 cells than in PURO and D141N cells (Figure 5)

We also observed a 3.4-fold and 3.8-fold reduction in FRAT1 (Frequently Rearranged in Advanced T-cell lymphomas 1) and 3-fold and 3.3 fold RIPK4 (Receptor Interacting Serine/Threonine Kinase 4) mRNA levels in the MCPIP1 cells relative to PURO and D141N cells (Figure 5). FRAT1 and RIPK4 modulate the Wnt signaling pathway, which is a crucial player in initiation and development of many cancers [101]. FRAT1 is a member of the GSK-3 (Glycogen Synthase Kinase 3) binding protein family, which selectively inhibits GSK3-mediated phosphorylation that is critical for stabilization and accumulation of β-catenin in cancer cells [102–106]. FRAT1 overexpression leads to aberrant activation β-catenin in many types of cancers [107–114]. Similarly, RIPK4 serine/threonine protein kinase overexpression has been reported in many cancers [115] and is attributed to aberrant activation of Wnt signaling [116]. Therefore, we postulate that MCPIP overexpression promotes growth inhibition and apoptosis in Caki-1 cells by downregulating FRAT1 and RIPK4, thereby decreasing Wnt signaling.

**Figure 6:**
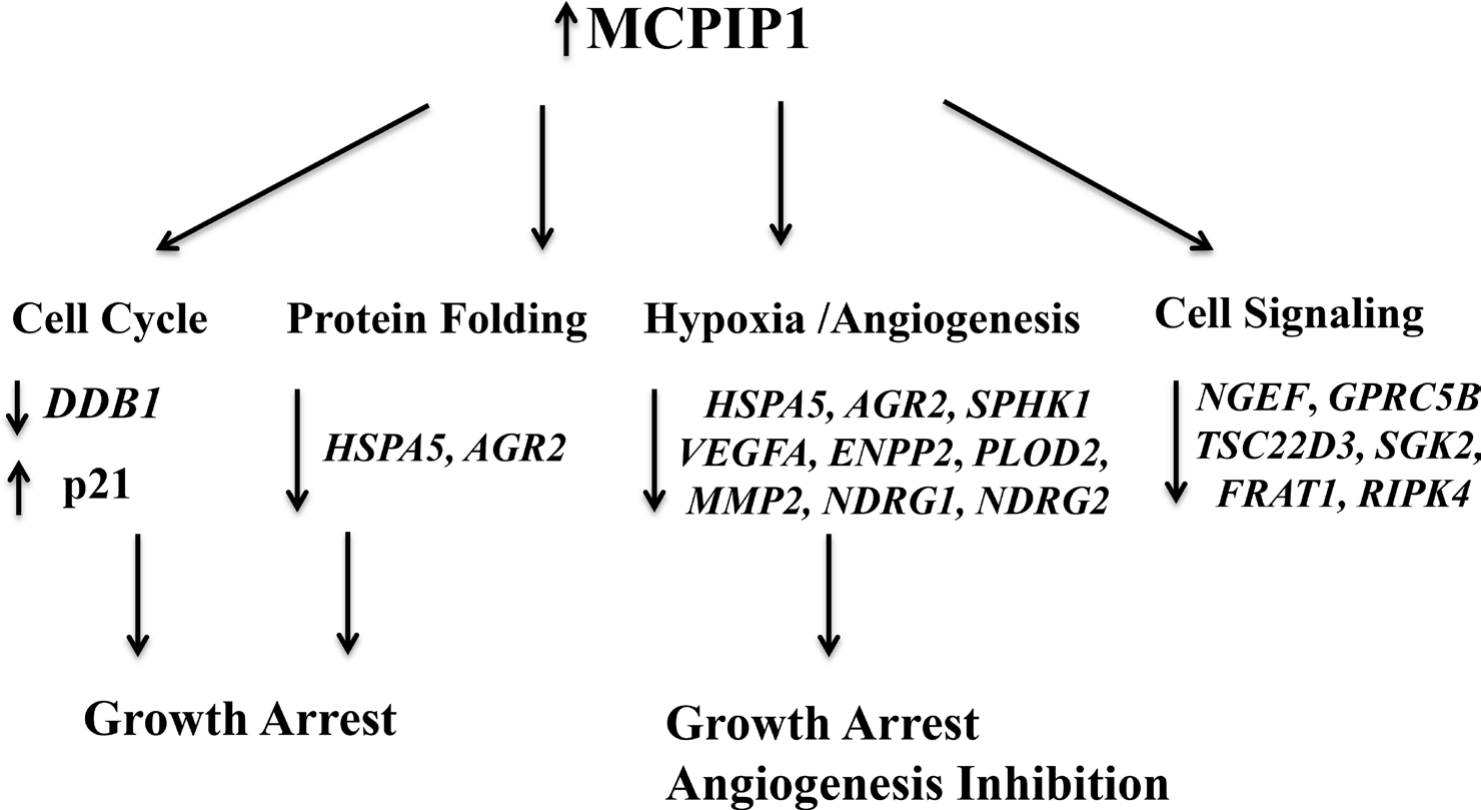
Schematic diagram shows transcriptome regulation by MCPIP1 in Caki-1 cells Detailed description is included in the Results and Discussion section.

In conclusion, we identified 219 transcripts that are downregulated in MCPIP1 cells, but are unchanged or upregulated in D141N cells that overexpress MCPIP1 that lacks the functional PIN domain. We further validated 15 selected transcripts by qRT-PCR. As shown in Figure 6, MCPIP1 overexpression leads to large scale changes in transcript levels, especially those involved in protein folding, cell cycle progression, hypoxia response, angiogenesis and cell signaling.

## MATERIALS AND METHODS

### Cell culture

The human ccRCC cell line, Caki-1, was cultured in Eagle minimal essential medium (EMEM; Lonza) containing 10% fetal bovine serum (FBS; BioWest) at 37°C and 5% CO_2_. Cells were passaged at 80–90% confluence, thrice a week.

### Transfection of Caki-1 with lentiviral constructs

We used the doxycycline-dependent TetON overexpression system for stable expression of wild type MCPIP1 and mutant form of MCPIP1 with inactivated PIN domain (D141N) (pLIX MCPIP1, pLIX D141N). Lentiviruses with the empty lentiviral vector (pLIX PURO) were transduced into Caki-1 cells to generate the negative control cells. Caki-1 cells were transduced with the lentiviruses carrying the different expression constructs at an MOI of 50 when they were 50–70% confluent. After 24 h, the virus containing media was replaced with fresh media for 24 h followed by selection in media containing 2 µg/ml puromycin (InvivoGen) for 10 days to select stably transduced cells.

### BrdU cell proliferation assay

Cell proliferation was measured with the BrdU Cell Proliferation Assay Kit (Roche). We seeded 5 × 10^3^ MCPIP1, D141N and PURO cells in 96-well plates and stimulated them with 1 µg/ml doxycycline (BioShop) for 24 h. Then, the cells were incubated with 10 μM BrdU for 8 h and the incorporated BrdU was quantified according to the manufacturer’s instructions by measuring the chemiluminescence with the Tecan Spectra Fluor Plus Microplate Reader (Tecan Group Ltd.). The experiment was performed four times and the data was presented as the mean luminescence value (percentage) for each sample relative to the PURO cells. Mean luminescence of the PURO cells was set as 100%.

### MTT cell viability assay

Cell viability was measured with the colorimetric MTT assay (Sigma). We seeded MCPIP1, D141N and PURO cells (5 × 10^3^ per well) in 96-well plates and stimulated them with doxycycline (DOX) for 24 h. Then, MTT assay was performed at 24, 48 and 72 h and measured the absorbance at 570 nm relative to 690 nm in a Tecan Spectra Fluor Plus Microplate Reader (Tecan Group Ltd.). The experiment was performed four times with quintuplicate samples in each experiment. Data was expressed as the mean absorbance of each sample relative to PURO cells.

### Statistical analysis

All statistical analyses were performed using GraphPad Prism (ver. 6.0). Statistical data is expressed as mean ± SD from at least three independent replicate experiments. Analysis of multiple datasets was performed by a one-way ANOVA analysis followed by Tukey’s HSD (honest significant difference) test. The significant *p*-values were marked as follows: ^*^ denotes *p* < 0.05; ^**^ denotes *p* < 0.01; ^***^ denotes *p* < 0.001; ^****^ denotes *p* < 0.0001.

### RNA isolation

Total RNA was isolated from stably transfected Caki-1 cells stimulated with doxycycline for 24 h with the guanidium isothiocyanate (GTC) method. Total RNA preparation was checked by 1% denaturing formaldehyde agarose gel electrophoresis for ribosomal RNA and DNA contamination. Total RNA concentration and its purity were assessed by determining the A260/280 and A260/230 ratios, respectively (NanoDrop).

### Generation of the transcriptome library and RNA sequencing

RNA integrity was assessed with the Agilent RNA 6000 Nano Kit on 2100 Bioanalyzer (Agilent) followed by library preparation with the Ion AmpliSeq Transcriptome Human Gene Expression Panel (Thermo) according to the manufacturer’s protocol [117]. Briefly, equal amounts of total RNA from all samples were reverse transcribed and the cDNAs were subjected to multiplex PCR to amplify parts of the target transcripts. The resultant amplicons were partially digested AND ligated with adapters. Ligation products were then purified with AMPure^®^ XP beads (Beckman). The library was quantified in the Bioanalyzer 2100 and the concentration was adjusted to ∼100 pM prior for template preparation. Then, eight barcoded library templates were clonally amplified on Ion Sphere particles (ISPs) with the Ion PI IC 200 Kit (Thermo) on the Ion Chef Instrument (Thermo Fisher Scientific, USA), loaded onto Ion PI chips and sequenced on an Ion Proton sequencer (Thermo) with the Ion PI IC 200 Kit, according to the manufacturer’s instructions.

### Analysis of RNA-sequence reads

The raw reads were processed by the Torrent Suite analysis pipeline and mapped to the human genome assembly hg19 AmpliSeqTranscriptome version by TMAP. The reads corresponding to each gene were counted with htseq-count [118]. The data was normalized and the differential expression of various genes was determined by DESeq2 using default parameters [119]. Overrepresentation of gene ontology (GO) terms was determined by the R/BioConductor package clusterProfiler with the hypergeometric test [120]. GO visualization was performed with the GOplot R package [121]. KEGG visualization was performed with the Pathview package [122]. The RNA-Seq data (Accession number: PRJEB20908) was deposited as BAM files in the European Nucleotide Archive (https://www.ebi.ac.uk/ena).

### Quantitative real-time PCR

We validated the selected transcripts by qRT-PCR. Total RNA was isolated from PURO, MCPIP1 and D141N cells from three independent experiments with the guanidium isothiocyanate (GTC) method as described.. Subsequently, 1 μg of total RNA was reverse-transcribed with oligo (dT15) primers (Promega) and M-MLV reverse transcriptase (Promega). The cDNA was diluted 5-times and real-time PCR was carried out using Eco Real-Time PCR System (Illumina) with the SYBR Green master mix (A&A Biotechnology) and primers that are listed in Supplementary Table 5. The relative levels of the transcripts were determined relative to RPS13 (ribosomal protein S13) by ΔΔCT method.

### Western Blot

Total protein lysates were prepared from MCPIP1, D141N and PURO cells and quantified with the bicinchoninic acid assay. Equal amounts of protein samples were resolved by SDS/PAGE on 10% Bis-Tris acrylamide gels at a constant voltage of 120V. The resolved proteins were then transferred to a PVDF membrane (Millipore) for 90 minutes at a constant voltage of 90 V. Then, the membranes were blocked with 5% non-fat milk in Tris-buffered saline containing 0.1% Tween-20 (TBST; BioShop) for 1 h at room temperature. Membranes were incubated with primary antibodies against MCPIP1 (1:2000; GeneTex 1:2000), p21 Waf1/Cip1 (12D1; 1:1000; Cell Signaling Technology) and β-actin (1:3000; Sigma), overnight at 4°C. Then, the membranes were incubated with HRP-conjugated secondary antibodies (anti-rabbit IgG or anti-mouse IgG, Sigma 1:30000). For signal detection, Immobilon Western HRP substrate (Millipore) was used and the chemiluminescence was determined in a ChemiDoc system (BioRad).

## SUPPLEMENTARY MATERIALS FIGURE AND TABLES










