# Supplementary material for: RNA sequencing reveals widespread transcriptome changes in a renal carcinoma cell line
Source: Oncotarget. 2018 Jan 16;9(9):8597–613. doi: 10.18632/oncotarget.24269 (PMC5823589; doi:10.18632/oncotarget.24269)
Supplement: Supplementary file 1 [file oncotarget-09-8597-s001.pdf]

RNA sequencing reveals widespread transcriptome changes in a renal carcinoma cell line

SUPPLEMENTARY MATERIALS

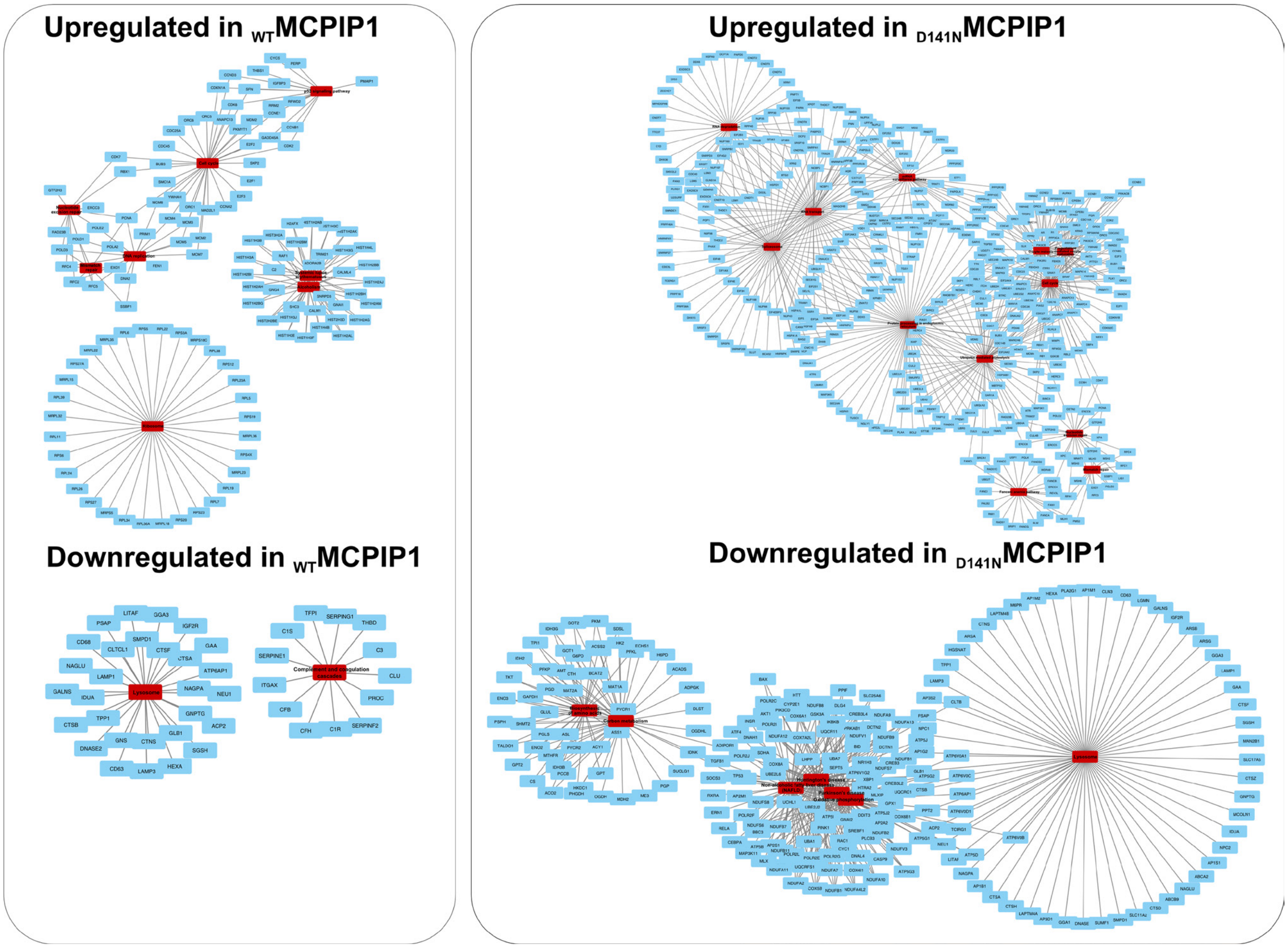

Supplementary Figure 1: Illustration of the interconnections between KEGG pathways for differentially regulated genes in MCPIP1 and D141N Caki-1 cells.

Supplementary Table 1: Gene expression list from RNA-Seq analysis. See Supplementary_Table_1

Supplementary Table 2: Top Gene Ontology (GO) terms and KEGG pathways representing differentially expressed transcripts in MCPIP1 vs. PURO group. See Supplementary_Table_2

Supplementary Table 3: Top Gene Ontology (GO) terms and KEGG pathways representing differentially expressed transcripts in D141N vs. PURO group. See Supplementary_Table_3

Supplementary Table 4: Top Gene Ontology (GO) terms and KEGG pathways representing differentially expressed transcripts in MCPIP1 vs. D141N group. See Supplementary_Table_4

Supplementary Table 5: List of primer sequences used in the study

Gene Symbol	NCBI Reference Sequence	Forward (5'->3')	Reverse (5'->3')	Product [bp]
RPS13	NM_001017.2	TCGGCTTTACCCTATCGACGCAG	ACGTACTTGTGCAACACCATGTGA	153
		**Cell Cycle**		
DDB1	NM_001923.4	TGCCTACAAGCCCATGGAAG	TGGCAGCGCTATCCTTTTGA	152
CDKN1A	NM_000389.4	CATGACAGATTTCTACCACTCCA	GGCCAGGGTATGTACATGAGG	200
		**Hypoxia/Angiogenesis**		
AGR2	NM_006408.3	AGGTGGGTGAGGAAATCCAG	GGGTTTGTTGCTTGTCTTGGA	240
ENPP2	NM_001040092.2	GCTGCAATAGCTCAGAGGACG	TTCAATGTCACGCACCCTAGC	80
HSPA5	NM_005347.4	TCAACATGGATCTGTTCCGGT	TTTGGAATTCGAGTCGAGCCA	121
MMP2	NM_001127891.1	ATTCCGCTTCCAGGGCACATCC	ACCAACAGTGGACATGGCGGTC	142
NDRG1	NM_001135242.1	GTGGAGGGCCTTGTCCTTATC	CATCCTGAGATCTTGGAGGCG	80
NDRG2	NM_201535.1	GCTTGCAGACATGATCCCTTG	TGTCCGGGTGGTTAAGAGCAT	116
PLOD2	NM_182943.2	ACGAAGGGATTTGCACTACTGA	AACCACCTCCCTGAAAGTCTTC	142
SPHK1	NM_021972.3	AGGCTGAAATCTCCTTCACGC	GTCTCCAGACATGACCACCAG	113
		**Cell Signaling**		
FRAT1	NM_005479.3	GTCCCAACCAGAAACCCGCA	TGATGAGGTTTCCAGAGAGCAC	80
GPRC5B	NM_016235.2	GGATGAACACAATGCAGCTCTC	TCTCAGTTGGCTGATACACGTT	116
NGEF	NM_019850.2	AGTTTGTTTCGTTCACATCCCG	CAAAGATCCACCCGTCGTCAG	147
RIPK4	NM_020639.2	ATGCCCACTACCACGTCAAG	TCTTCTCATCTGCAAACGGCT	228
SGK2	NM_170693.2	TTCAACCCAAATGTGACAGGACC	AATGGACTTGGACACAGCTTC	81
TSC22D3	NM_198057.2	ATAATGCGGCAGGATTCGCTA	GGAGGCACTGTGGAAGAAGAG	126
